# An Ancient Relation between Units of Length and Volume Based on a Sphere

**DOI:** 10.1371/journal.pone.0033895

**Published:** 2012-03-28

**Authors:** Elena Zapassky, Yuval Gadot, Israel Finkelstein, Itzhak Benenson

**Affiliations:** 1 Institute of Archaeology, Tel Aviv University, Ramat Aviv, Tel Aviv, Israel; 2 Department of Geography and Human Environment, Tel Aviv University, Ramat Aviv, Tel Aviv, Israel; Illinois State University, United States of America

## Abstract

The modern metric system defines units of volume based on the cube. We propose that the ancient Egyptian system of measuring capacity employed a similar concept, but used the sphere instead. When considered in ancient Egyptian units, the volume of a sphere, whose circumference is one royal cubit, equals half a *hekat*. Using the measurements of large sets of ancient containers as a database, the article demonstrates that this formula was characteristic of Egyptian and Egyptian-related pottery vessels but not of the ceramics of Mesopotamia, which had a different system of measuring length and volume units.

## Introduction

Knowledge of the connection between linear dimensions and volume of containers is important, for instance, in order to achieve quick estimates of trade commodities. However, in many measuring systems, both ancient and modern, the length and volume units seem to have emerged independently, without a simple, intrinsic relation between them [1]. One of the advantages of the metric system, initiated at the time of the French Revolution, is the introduction of such a relationship: 10 cm^3^ make 1 liter [2], meaning that the unit of volume is based on the unit of length, employing a cube as an elementary body. For the sake of simplicity, we do not distinguish in this paper between the notions of volume and capacity and use “volume” throughout. This conceptually convenient definition, however, does not help in practical measurements, as most containers are not cube-shaped.

The use of the cube of a length-unit edge can be traced in antiquity in ancient Egypt. The Egyptian unit of length and volume were the royal cubit and *hekat*. Various pieces of evidence – papyri, inscribed vessels and monumental texts – attest to the *hekat* as the dominant unit in practical activities, e.g., in measuring stored grain and liquids [3], [4]. According to the evidence of ancient rods and marked vessels, the royal cubit is estimated as ∼52.3 cm, and consists of 28 smaller units called fingers. The *hekat* is estimated as ∼4.8 liters [3], [4]. Ceremonial stone cubit rods were kept in temples and were considered as possessing spiritual meaning: the inscription on the rods described in [5] says “The cubit is life, prosperity, and health, the repeller of the rebel …”. A similar statement can be found on the wooden cubit rod in [6]: “… [Gods]… may give life, prosperity, and health, and good lifespan …”

The cube of one cubit edge was used in ancient Egypt for estimating soil volumes in earthworks, see [7] for construction account in Papyrus Reisner I, Section I, and the Egyptians knew how to convert cubits into *hekats*. Translating Problems 41 and 44 in the Rhind Papyrus [3], [7], [8], [9] to modern mathematical formulae, one learns that the volume of a cube of 1 cubit-edge equals 30 *hekats*, i.e., (1 royal cubit)^3^/30 = 1 *hekat*. Using the value of 1 royal cubit = 52.3 cm, one indeed obtains, according to the above-mentioned Rhind Papyrus problem, an estimate of one *hekat* = 4.77 liters.

**Figure 1:**
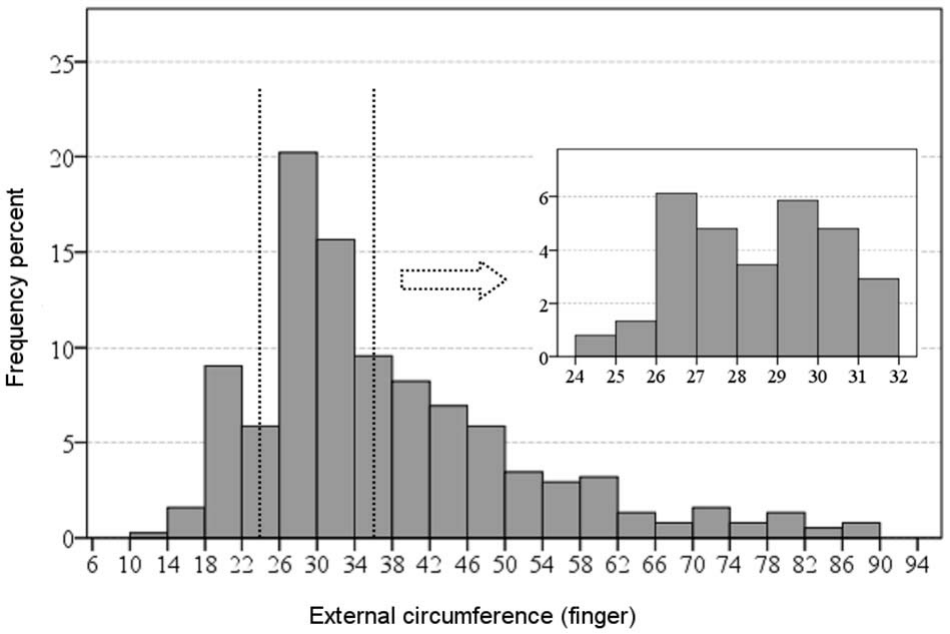
The circumference of 376 Egyptian New Kingdom ovoid-shaped jars presented in three recent publications [**10]**–[12****]. According to dip test (see methods below) the distribution is unimodal, p = 0.66.

This cube-based relation was of little use in the typically ovoid-shaped Egyptian ceramic jars [10]–[12]. Surprisingly, our measuring of the circumference of hundreds of Egyptian ovoid-shaped jars according to their drawings demonstrates preference for vessels whose maximal external horizontal circumference varies between 26–32 fingers, i.e., 1 cubit±2 fingers (see Fig. 1).

Can the knowledge that the circumference of an ovoid-shaped container is 1 cubit assist in estimating its volume? Below we present evidence that the inherent relationship between ancient Egyptian units of length and volume measurements can be based on another elementary body – the sphere – and test our hypothesis based on the available archaeological information. We also demonstrate that the revealed relation was not relevant in Mesopotamia, where a different system of measuring length and volume units was in use.

## Results and Discussion

In Egyptian units of length and volume, the volume of a spherical container of 1 cubit circumference would be 0.5 *hekat*. Indeed, the volume v of a sphere of a circumference c equals:
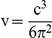



Substituting 1 royal cubit for c and employing the above-mentioned solution to Problems 41 and 44 in the Rhind Papyrus, one obtains
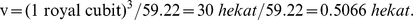



**Figure 2:**
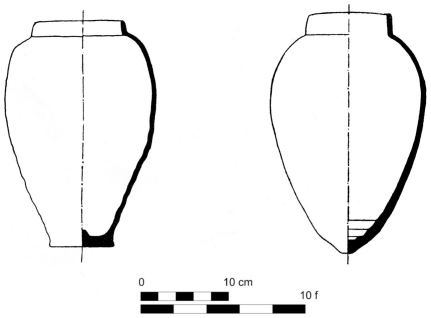
Typical Egyptian beer jars [**13, Plate IX b, jars 28 and 24**].

We checked the 1 royal cubit circumference
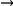
½ *hekat* relation in several available sets of Egyptian and Egyptian-related ceramic containers. First, we opted for a large set of New Kingdom Egyptian ovoid-shaped beer jars [10]–[14] (Fig. 2).

**Figure 3:**
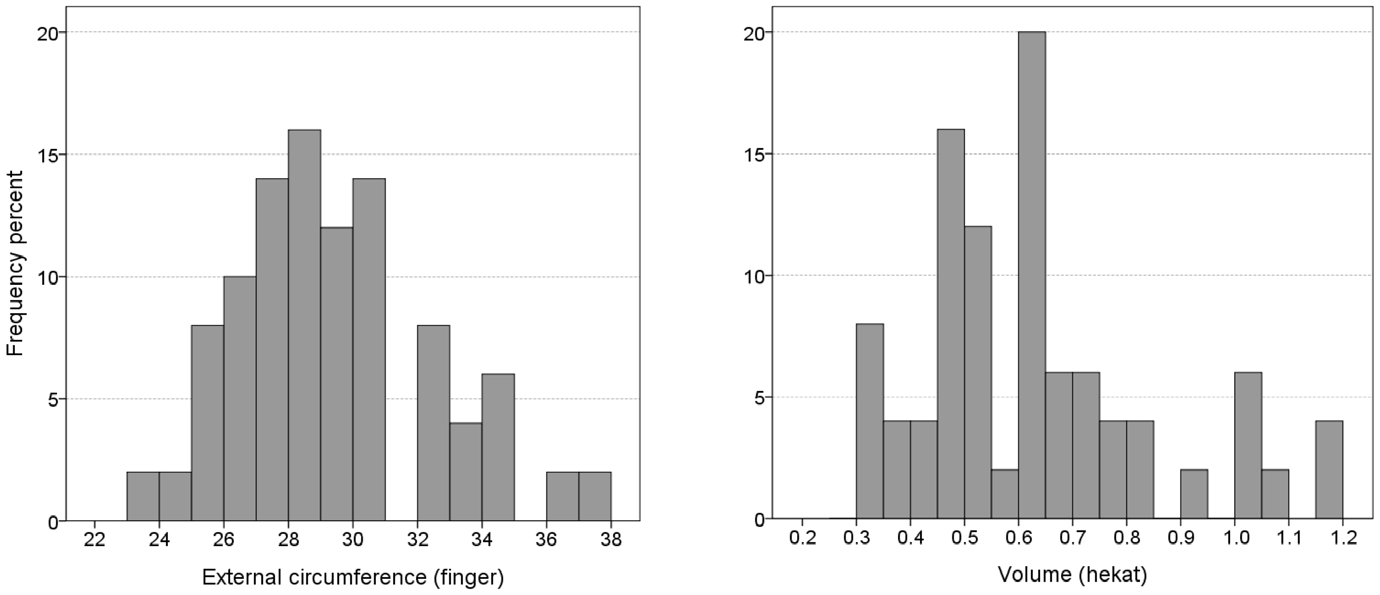
Maximal external circumference (a) and internal volume (b) of 50 typical Egyptian beer jars, drawings in [**10]**–[14****]. According to the dip test both distributions are unimodal: p = 0.70 for the circumference and p = 0.43 for the volume.

Despite variation in size, the most frequent maximal external circumference of these vessels (measured by us according to their drawings) indeed varies between 27 and 31 fingers (i.e., slightly above 1 royal cubit) and their modal volume, accounting for a wall width of 0.5–1.5 cm, varies between 0.45–0.65 *hekat* (Fig. 3).

Similar modal circumference and volume values were revealed by Barta [15], who studied 39 beer jars from the Old Kingdom site of Abusir. According to his data, one can estimate that the jars from the temple of Raneferef and the tomb of Fetekta fit our hypothesis; their volume vary between 1.9 and 2.6 liters (0.39–0.54 *hekat*), with the mode at 2.4 liters (0.50 *hekat*), while their circumferences vary between 47 and 57 cm (25.2–30.2 fingers). The beer jars found in the tomb of Kaaper are smaller though they have almost identical volume – ca 1.5–1.6 liters (0.31–0.33 *hekat*) – and their modal circumferences vary between 43.9 and 47.1 cm (23.5–25.2 fingers). Barta [15] argued that the jars were used as a unit for daily rations of food/beer.

Beer jars were produced in the coiling technique [15], [16] and the method of production can be related to the maximal circumference of 1 cubit: the potter started building the jar from its base, but could have prepared the longest coil of 1 cubit in advance, to be used in the middle of the vessel.

Globular pottery vessels – the best to demonstrate the 1 royal cubit circumference→½ *hekat* relation – are not common in Egypt proper. We therefore turned to perfect sphere-shaped ceramic jugs produced in late Iron Age I (ca. 1000 BCE) Phoenicia. We think that it is legitimate to do so because of the long-lasting tradition of cultural connections between Phoenicia and Egypt [16], which commenced as early as the third millennium BCE and continued until at least the 8^th^ century BCE [17]–[21]. This influence can be observed in different realms such as pictorial representations on seals and seal impressions [22], art representations [23], [24] and pottery production [25].

**Figure 4:**
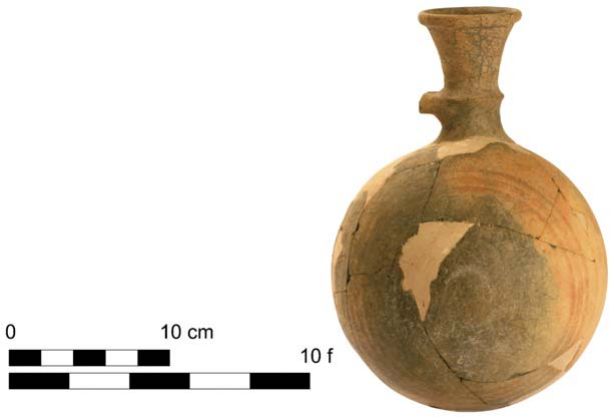
A Phoenician globular jug from Megiddo, with a maximal external circumference of 29.2 fingers and volume of 0.53 *hekat*.

We examined 89 Iron Age I-IIA Phoenicia-made globular jugs. Three of them we measured manually: one jug from Megiddo in the Jezreel Valley (Fig. 4) and two jugs from Tel Masos in the Beer-Sheba Valley. The other 86 jugs were measured according to their drawings; 55 come from Cyprus [26], [27], seven from Tyre [28] and 25 from various locations in Israel: Megiddo, Tel Dor, Tel Keisan, Hazor, Tell Qasile, etc. [29]–[36].

**Figure 5:**
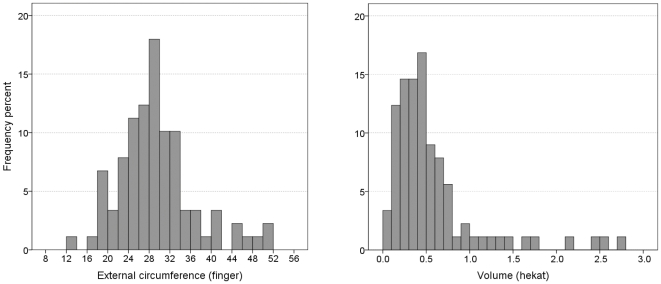
The circumference (a) and calculated volume (b) of 89 Phoenician globular jugs [**26]**–[36****]. According to the dip test both distributions are unimodal: p = 0.83 for the circumference and p = 0.72 for the volume.

In this case, too, the distribution of the jugs' external maximal circumference has a clear mode at 25–30 fingers (Fig. 5a). Taking into consideration a wall width of 0.5–0.7 cm, they provide a modal volume of 0.5 *hekat* (Fig. 5b).

It is possible that the Phoenician globular jugs were used in trade of valuable liquids [34]. The inherent relationship between the royal cubit and the *hekat* could have made a quick estimate of their capacity possible.

**Figure 6:**
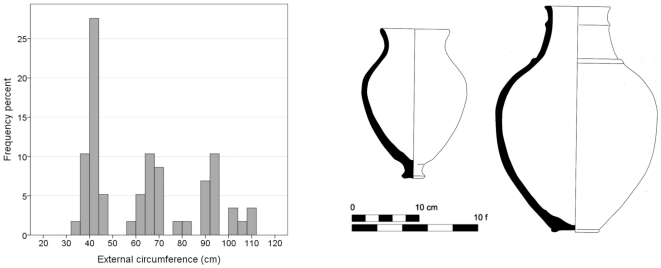
The circumference of 58 Late Bronze jars from Tell Sabi Abyad in north Syria [**37**] (a); Typical Late Bronze jars from Tell Sabi Abyad [**37, Figure IV.108, jars c and n**] (b). According to the dip test the hypothesis that the distributionis unimodal can be rejected at p = 0.06.

In order to establish whether the sphere-based relation of 1 royal cubit circumference
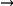
½ *hekat* was not just a coincidental expression of ovoid-shaped containers of that size being convenient for daily use, we turned to ovoid-shaped vessels in Mesopotamia. We analyzed the circumference and volume of 58 Late Bronze jars from Tell Sabi Abyad [37] in north Syria. Here too the analysis was performed according to their published drawings. It revealed three size groups, none featuring 52–53 cm circumference (proxy of a royal cubit), or 2.4 liters volume (proxy of 0.5 *hekat*) characteristic of the Egypt-related jars. To the contrary, there is a clear gap in these values in the jars' volume distribution (Fig. 6). This means that despite certain similarities in the use of volume units in Egypt and Mesopotamia, the different units in the latter did not result in a similar, straightforward relationship between units of length and volume that is based on a sphere.

**Figure 7:**
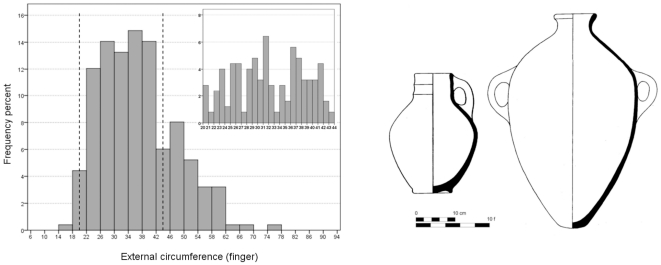
The circumference of 249 Late Bronze ovoid-shaped jugs and jars found at Megiddo [**38**] (a); Typical Late Bronze ovoid-shaped jug (left) and jar (right) from Megiddo [**38, Plate 33, jug 5 and jar 8**] (b). According to the dip test the hypothesis that the distribution of the circumference is unimodal can be rejected at p = 0.11.

One could have expected that the use of such formulae would have started in the Late Bronze Age, when the Levant, including Phoenicia, fell under direct Egyptian sway [19]. However, our study of ovoid-shaped Late Bronze jugs and jars from Megiddo [38] (Fig. 7) provides the modal interval of the circumference as 22–42 fingers, that is essentially wider than the modal interval of the circumference of the Egyptian jars, 26–32 fingers (Fig. 1). Although most of the complete vessels chosen for the comparison were found in tombs, they well-represent the daily, domestic repertoire at Megiddo. Looking at the modal interval of the distribution of the Megiddo jugs and jars' circumference at higher a resolution (inner histogram in Fig. 7) makes it possible to assume that it has more than one mode; one of the modal intervals is 25–32 fingers, the same as that of the Egyptian jars. However, the dip statistical test (p = 0.11) does not allow for definite conclusion. From a broader perspective, it is questionable whether at that time the Phoenician cities had achieved a commercial status similar to what they had in the later Iron Age. Moreover, the very fact that globular vessels are not frequent in Phoenicia in the Late Bronze Age seems to indicate that the idea of connection between the circumference of the globular jar and its volume developed later.

The ancient Egyptian 1 royal cubit
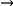
½ *hekat* relation in a sphere, detected in pottery vessels, sheds light on the practice of daily measurements of volume of liquids in the Ancient Near East. We have discovered this relation based on the analysis of the form and volume of a large number of Egyptian and Phoenician jars. Phoenician globular jars best express this relation: their circumference concentrates around the value of 1 cubit, while their volume is around ½ *hekat*. What is missing in order to confirm our discovery is textual evidence which would discuss the relation between circumference and volume in ovoid-shaped jars.

To conclude, the ancient Egyptian 1 royal cubit
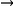
½ *hekat* relation in a sphere is no less sophisticated than the modern 10 cm^3^ 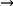
1 liter relation expressed in a cube. This wisdom of sphere-based relationship, which was inherent and possibly unique to Egypt and its cultural sphere of influence, was lost over the ages.

## Materials and Methods

The external circumference of a jar was estimated by direct measurement or by multiplying the length of the widest horizontal cross-section of a drawing by π.

In order to estimate the volume of a jar we scan the drawing, digitize its external and internal contours, and construct a 3D model by rotating its internal and external contours with Rhinoceros™ software. We can then estimate the volume of the jar, up to the neck, according to the internal contour. We estimate the wall width according to the drawings as well as by manually measuring the volume of three of the jugs and compared the result to the estimates obtained according to the digitized external profile. As we have demonstrated elsewhere [39], in the case of a symmetrical jar, this procedure provides an adequate estimate of its volume.

The unimodality of the distribution was tested according to the dip test [40]. We employed the MATLAB software provided by [41], which implements the algorithm of [42] and applies bootstrapping for significance estimation.
